# Leveraging Social Media Data to Understand the Impact of COVID-19 on Residents' Dietary Behaviors: Observational Study

**DOI:** 10.2196/51638

**Published:** 2025-05-23

**Authors:** Chuqin Li, Alexis Jordan, Yaorong Ge, Albert Park

**Affiliations:** 1 Department of Software and Information Systems, College of Computing and Informatics University of North Carolina Charlotte, NC United States; 2 School of Data Science, College of Computing and Informatics University of North Carolina Charlotte, NC United States

**Keywords:** social media, obesity, COVID-19, dietary pattern, observational study, dietary behaviors, Twitter, tweets, eating patterns, food consumption, social media data

## Abstract

**Background:**

The COVID-19 pandemic has inflicted global devastation, infecting over 750 million and causing 6 million deaths. In an effort to control the spread of the virus, governments around the world implemented a variety of measures, including stay-at-home orders, school closures, and mask mandates. These measures had a substantial impact on dietary behavior, with individuals discussing more home-cooked meals and snacking on social media.

**Objective:**

The study explores pandemic-induced dietary behavior changes using Twitter images and text, particularly in relation to obesity, to inform interventions and understand societal influences on eating habits. Additionally, the study investigates the impact of COVID-19 on emotions and eating patterns.

**Methods:**

In this study, we collected approximately 200,000 tweets related to food between May and July in 2019, 2020, and 2021. We used transfer learning and a pretrained ResNet-101 neural network to classify images into 4 health categories: definitely healthy, healthy, unhealthy, and definitely unhealthy. We then used the state obesity rates from the Behavioral Risk Factor Surveillance System (BRFSS) to assess the correlation between state obesity rates and dietary images on Twitter. The study further investigates the effects of COVID-19 on emotional changes and their relation to eating patterns via sentiment analysis. Furthermore, we illustrated how the popularity of meal terms and health categories changed over time, considering varying time zones by incorporating geolocation data.

**Results:**

A significant correlation was observed between state obesity rates and the percentages of definitely healthy (*r*=–0.360, *P*=.01) and definitely unhealthy (*r*=0.306, *P*=.03) food images in 2019. However, no trend was observed in 2020 and 2021, despite higher obesity rates. A significant (*P*<.001) increase in the percentage of healthy food consumption was observed during (39.99% in 2020) and after the shutdown (39.32% in 2021), as compared with the preshutdown period (37.69% in 2019). Sentiment analysis from 2019, 2020, and 2021 revealed a more positive sentiment associated with dietary posts from 2019. This was the case regardless of the healthiness of the food mentioned in the tweet. Last, we found a shift in consumption time and an increase in snack consumption during and after the pandemic. People ate breakfast later (ie, from 7 AM to 8 AM in 2019 to 8 AM to 9 AM in 2020 and 2021) and dinner earlier (ie, from 6 PM to 7 PM in 2019, to 5 PM to 6 PM in 2020). Snacking frequency also increased. Taken together, dietary behavior shifted toward healthier choices at the population level during and after the COVID-19 shutdown, with potential for long-term health consequences.

**Conclusions:**

We were able to observe people’s eating habits using social media data to investigate the effects of COVID-19 on dietary behaviors. Deep learning for image classification and text analysis was applied, revealing a decline in users’ emotions and a change in dietary patterns and attitudes during and after the lockdown period. The findings of this study suggest the need for further investigations into the factors that influence dietary behaviors and the pandemic’s implications of these changes for long-term health outcomes.

## Introduction

### Overview

The COVID-19 pandemic is a global pandemic declared by the World Health Organization on March 11, 2020, that has had a devastating impact on people and economies around the world. As of January 2022, more than 750 million people have been infected and more than 6 million people have died from COVID-19 worldwide [1]. On March 13, 2020, a national emergency was declared in response to the COVID-19 pandemic in the United States. In the wake of the declaration, many states began to issue stay-at-home orders in an effort to slow the spread of the virus. In addition to stay-at-home orders, many states also implemented other measures, including the closure of public schools, announcement of mask mandates, and limitations on restaurant capacities to curb the spread of COVID-19. These disruptions have fundamentally altered people’s daily routines, inducing significant lifestyle changes with potential long-term effects on public health.

Efforts to counteract the spread of COVID-19 had a significant impact on people’s lifestyles and dietary behavior. One of the most significant changes includes purchasing more food per grocery shopping trip due to the desire to avoid crowded stores and restrictions on restaurants [2,3]. This led to preparing more meals at home, which can potentially have an impact on long-term food preferences. For example, researchers used data from NutriQuébec, a cohort study designed to track changes in adult Quebecois dietary habits over time. They compared questionnaire responses between June 2019 and February 2020 to responses from early lockdown (April to May 2020). They measured diet quality using the Healthy Eating Index 2015 and found that adults aged between 18 and 29 years, adults with lower education, and adults who were obese showed increases in Healthy Eating Index 2015 during early lockdown. They attributed this to small improvements in the intake of whole grains, greens, beans, refined grains, total vegetables, total dairy, seafood, plant proteins, added sugar, and total protein [4].

Psychological stress has also been associated with increased consumption of food, especially high-fat foods [5]. Additionally, food insecurity increases the probability of low-income families consuming easily accessible, highly palatable foods that are high in fat and sodium [6], which can lead to the development of obesity. The COVID-19 shutdown has had a significant impact on people’s dietary behaviors. It is important to investigate these changes to understand their impact on people’s health and well-being.

In this study, we aim to investigate the impact of the COVID-19 shutdown on population-level dietary behavior, particularly in relation to obesity. By understanding how these lifestyle changes have influenced dietary behaviors, we can provide predictive value for public health; first, it helps public health authorities and policy makers to gauge the impact of pandemic-related interventions; second, it provides insights into how behaviors related to diet may evolve in response to future crises. This understanding will help us develop more effective public health strategies and interventions to mitigate negative consequences associated with pandemics in the future [7], particularly concerning obesity and other diet-related health issues.

To achieve this, we collected and analyzed a large pool of images posted by individuals on the popular social media platform, Twitter. Twitter is one of the most diverse social media platforms in terms of user age [8]. Given that Twitter has limited contextual information due to tweet length restrictions and the increasing number of multimedia postings [9], our study uses text with image data to learn peoples’ dietary behaviors to learn about population-level dietary behavior in a more comprehensive way. Specifically, we aim to identify patterns and trends in food choices and consumption habits at the state level during different phases of the pandemic: before, during, and after the shutdown. Our study will provide insights into the complex interplay between social and environmental factors that can shape people’s obesity-related dietary behavior and inform the development of targeted interventions to promote healthier eating habits and combat obesity.

### Background

Studies examining various COVID-19–related lifestyle changes have been conducted. The conclusions of the studies depend on their time frame, participants’ demographics, and data collection and analysis methods. For example, Cosgrove and Wharton [10] used survey data to examine perceived changes in dietary healthfulness of the general US population during the COVID-19 pandemic. They found dietary habits were perceived to become healthier. In contrast, Rogers et al [11] conducted a longitudinal survey study between March 30 and April 7, 2020, and 8 months later between November 2 and November 21, 2020. They found that dining out and consuming take-out and fast food increased, and the consumption of frozen meals decreased among respondents from April to November based on 636 respondents who completed surveys throughout the duration of the study. Rogers et al’s [11] study examined the early behavioral changes at the onset of the pandemic, while Cosgrove and Wharton [10] observed longer-term adaptations. In addition, these 2 studies have a significant difference between participants’ demographics and socioeconomic factors. Compared with these 2 studies, our study has a longer time period, a wider demographic population, and does not rely on survey data. With regards to physical activity, Curtis et al [12] surveyed 61 parents in Adelaide, Australia, and found that participants slept longer, got up later, and spent less time engaging in light physical activity.

The constant barrage of COVID-19 news can lead to increased stress levels, which can trigger stress- and fear-eating in some people [13]. People who experience fear, anxiety, or stress often turn to “comfort food” or snacks as a way to cope, which are typically high in sugar and fat [14,15].

Although surveys provide high-quality and targeted data, they are costly and can be limited in sample size. Social media, on the other hand, is widely used in the United States [16], and data from social media is generally free to collect. Social media data can also provide insights more rapidly and comprehensively [17]. Park et al [18] summarized a growing body of literature and illustrated the successful use of social media for health. Li et al [19] proposed an approach that predicts state-level obesity rates using social media data. Users’ obesity-related behaviors in social media were found and could be classified into 4 levels of interaction: individual, interpersonal, web-based social environment, and connection to the real world [20]. Additionally, the use of social media platforms increased by 61% during the pandemic [21]. Social media can impact how we study lifestyle diseases, such as obesity, because an increasing number of people’s lives are shared via the web [22]. In the last couple of years, social media users have shifted from text only to multimedia posts, such as images and text [23]. Social media image data could contain additional information that is not provided in the textual data [24], since uploading images is easier and faster with a smart mobile device.

We collected Twitter data from the months of March to June in 2019, 2020, and 2021 to investigate residents’ food behavior before, during, and partially after the shutdown. We investigate how the COVID-19 shutdown impacted state-level dietary behavior and the relationships between residents’ food behavior and the state-level obesity rate. Image classification models, text analysis methods, and visualization tools are used to answer 2 research questions in this study:

- RQ1: Does the state obesity rate correlate with the dietary images shared on Twitter?
- RQ2: How has the COVID-19 shutdown affected people’s dietary behavior?

## Methods

### Data Collection

Twitter has a broad demographic user base across ethnicity, gender, age, and income [25]. This makes it a good source of data to learn users’ dietary behaviors on a large scale with fewer biases compared with niche social media platforms. We used the Twitter public streaming application programming interface [26] to collect tweets about food and their associated metadata (eg, temporal, geolocation, and user information). We only collected tweets with images containing hashtags related to meals (Table 1) based on a previous study [27]. We collected approximately 200,000 tweets from May, June, and July of 2019, 2020, and 2021. Tweets are short posts limited to 140 characters [28]. In our collection, the average length of tweets was 13.3 words, after filtering out URLs, nonalphanumeric characters, special characters, and punctuation. The tweet collection contains around 220,000 unique words.

**Table 1:** Number of tweets we collected per hashtag from May, June, and July 2019, 2020, and 2021. The inclusion criteria were that the tweet needs to have geolocation information and images.

Hashtags	Year, n (%)		
	2019 (n=83,970)	2020 (n=68,429)	2021 (n=57,151)
#Breakfast	17,203 (20.49)	12,956 (18.93)	10,728 (18.77)
#Lunch	25,157 (29.96)	16,855 (24.63)	15,629 (27.35)
#Dinner	25,742 (30.66)	23,431 (34.24)	18,597 (32.54)
#Brunch	5956 (7.09)	3471 (5.07)	4376 (7.66)
#Snack	3207 (3.82)	3247 (4.75)	2426 (4.24)
#Meal	5631(6.71)	7421 (10.84)	4684 (8.20)
#Supper	863 (1.03)	913 (1.33)	615 (1.07)

### RQ1: Does the State Obesity Rate Correlate With the Dietary Images Shared on Twitter?

**Figure 1:**
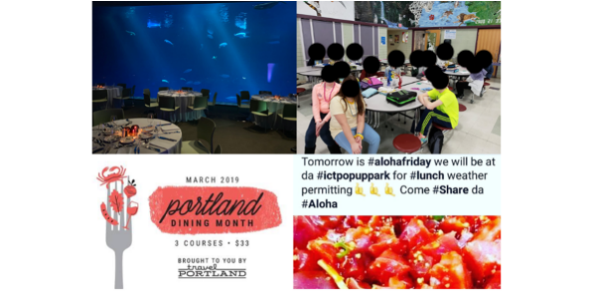
Example of nonfood images.

We used image classification techniques to examine the healthiness of food choices by classifying food images posted on tweets into 4 different health categories: definitively unhealthy, unhealthy, healthy, and definitively healthy based on a previous study [29]. This healthiness classification schema was developed by Vydiswaran et al [30]. In their study, public health nutritionists assess the healthfulness of a list of food-related keywords by categorizing them into 4 distinct health categories. This list served as the foundation for our dataset, which contains a mix of food and nonfood images, despite their tagging. People tweet images about not only what they eat but also who they eat with and where they eat. Examples of nonfood images are shown in Figure 1. Because of this, we designed a workflow to categorize images to healthiness. We first classify images into food and nonfood (food classification), and then classify food images into different health categories (health category classification).

For the 2 proposed classification tasks, we used transfer learning. We used a pretrained ResNet, which was trained on more than a million images from the ImageNet database. We selected ResNet-101, a deep convolutional neural network architecture based on ImageNet, for its effectiveness in large-scale image classification [31]. ResNet-101 addresses the vanishing gradient problem, a major challenge in deep networks, by introducing skip connections. These connections allow the network to learn from both the current layer and earlier layers, improving performance compared with other architectures, such as very deep convolutional (visual geometry group) networks [32]. Similarly, ResNet-101 provides a balance between simplicity and scalability compared with DenseNets [33]. Our choice of the 101-layer variant is motivated by its computational efficiency while maintaining performance similar to deeper versions like ResNet-152. Image features learned from the pretrained ResNet-101 are transferred to a SoftMax layer for final classification tasks. To feed image data into the deep learning model, we preprocessed our image data by cropping each image to 224 *×* 224. We used the Adam optimizer and categorical cross-entropy as the loss function.

We used the publicly available Food-5K dataset [34] as the training data to train the food classification model to classify images into food and nonfood. The Food-5K dataset contains 2500 food and 2500 nonfood images. The food images were selected from existing and publicly available food image datasets and visually inspected and categorized by human observers into 2 classes: food and nonfood [34]. To train the health category classification model, we classified images into 4 different health categories, following the study from Vydiswaran et al [30]. We used Google image search to collect the training dataset by searching and downloading images for each food item. The total number of images we collected was 23,151. We split the training datasets, 80-20, for training and validation. We fine-tuned the parameters of pretrained ResNet-101 in the training process. We used the fine-tuned classification model with the lowest validation loss to classify images that we collected from tweets to different health categories. We used the percentage of healthy/unhealthy food in dieting images on Twitter at the state level to study the relationship between the state obesity rate and dieting images on Twitter.

### RQ2: How Has the COVID-19 Shutdown Affected People’s Dietary Behavior?

In our RQ2, we examine how the COVID-19 shutdown impacted people’s emotions and eating behaviors, potentially leading to increased unhealthy eating and weight gain. Specifically, we examined how COVID-19 affected dietary behavior, such as the time/frequency of each meal and how often healthy/nonhealthy foods were consumed.

Because tweets are short text and food-related tweets contain some food-specific sentiment words, the traditional dictionary-based sentiment analysis method, like Linguistic Inquiry and Word Count may not be applicable [30]. We used the Valence Aware Dictionary for Sentiment Reasoning (VADER) to conduct the sentiment analysis. VADER is a parsimonious rule-based sentiment analysis model, especially attuned to social media contexts [35]. VADER recognizes slang and emojis and appropriately adjusts the intensity of the sentiment score by combining signals. A positive, neutral, negative, and compound sentiment score was given to each tweet. The compound score is a measure of sentiment that is calculated by summing the valence scores of each word, adjusting according to the rules, and then normalizing to be between –1 and 1. The –1 represents the most extreme negative, and 1 is the extreme positive. In this study, we used the compound score of each tweet for the sentiment analysis. We used the threshold value 0 to classify tweets into different sentiment categories. If the compound score of a tweet was smaller than 0, we classified this tweet as negative; otherwise, this tweet was classified as positive.

Temporal histograms allow us to see how popular terms change over time. We visualized the popularity of “breakfast,” “lunch,” “dinner,” and “snacks” at the granularity of hours in a day. Additionally, we also summarized the hourly change of the distribution of the 4 food health categories of food in a day. To accommodate the different time zone issues, we first converted all tweets’ post time to their local time based on location. To identify the location of each tweet, we use the geolocation data we collected with Twitter application programming interface. Tweet location can be an exact “point” location or a Twitter Place with a “bounding box” that describes a larger area ranging from a venue to an entire region [36]. If a tweet contains an exact location, we use that information to change it to its local time. For tweets with a “bounding box,” we calculated the latitude and longitude of the center point for that box and used that information to change to its local time. In addition to how the COVID-19 shutdown changed dietary behaviors, we also examined what healthy/nonhealthy food was consumed by users before, during, and after the COVID-19 shutdown.

### Ethical Considerations

We only analyzed publicly available documents in this study and did not analyze identifiable private information or involve any direct or indirect interactions with individuals. Per UNC Charlotte and staff policy, the study is exempt from institutional review board requirements because it does not meet the regulatory definitions of human subjects research. Additionally, we removed any user-identifiable information (eg, usernames).

## Results

### RQ1: Does the State Obesity Rate Correlate With the Dietary Images Shared on Twitter?

We used the saved classification models to classify the tweets’ images. The accuracy of the food classification model on the validation data was 95.36%, and the accuracy of the health category classification model on the validation data was 85.21%. We further evaluated the saved models on our Twitter image data. We manually selected 200 images and labeled them. The accuracy of the food classification model on the Twitter image data was 90.00%, and the detailed performance of food classification models is shown in Table 2. The model has higher recall than precision for images containing food items. The primary reason an image containing food items could be incorrectly predicted as an image without food items is that we only cropped the center of the image, but the food is at the corner of the picture. We incorrectly predicted nonfood images as images containing food because images with McDonald’s and Burger King logos are classified as images containing food by the model.

Overall, the health category classification model was less accurate on Twitter image data than on the training dataset. The accuracy for the health category classification model on Twitter image data was 63.64%. The performance of the food classification model for all the classes individually is shown in Table 3.

**Table 2:** Image classification model performance.

Class	Precision	Recall	*F*_1_-score	Number of occurrence
No food	0.88	0.97	0.92	118
Contain food	0.94	0.80	0.87	82

**Table 3:** Health category classification model performance.

Class	Precision	Recall	*F*_1_-score	Number of occurrence	Predicted definitely healthy	Predicted healthy	Predicted unhealthy	Predicted definitely unhealthy
Definitely healthy	0.64	0.60	0.62	15	9	2	2	2
Healthy	0.58	0.54	0.56	13	2	7	1	3
Unhealthy	0.87	0.63	0.73	32	3	3	20	6
Definitely unhealthy	0.35	1	0.52	6	0	0	0	6

**Figure 2:**
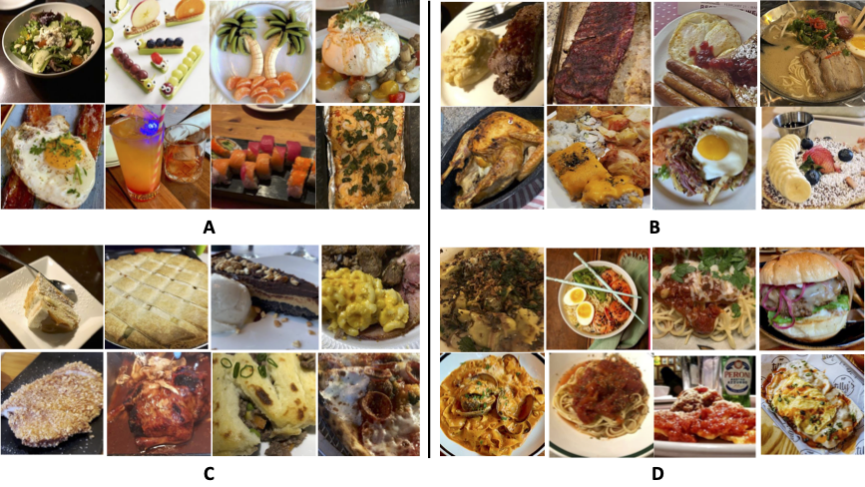
Performance of the image classification model on food healthiness predictions. (A) Food in these images is predicted as definitely healthy. (B) Food in these images is predicted as healthy. (C) Food in these images is predicted as definitely unhealthy. (D) Food in these images is predicted as unhealthy.

Figure 2 shows the prediction of food images in their respective classes. The top left images in Figure 2 contain images of salad, fruit, sushi, cheese, and poached egg, which are classified to represent a category of definitely healthy. The food images are classified to represent the category of definitely unhealthy is shown in the bottom left. These include images for cake, pie, ice cream, chocolate, mac and cheese, and some meat. The top right is the food images classified as healthy. This class includes images of roast chicken, egg, pancake, mashed potato, etc. Images on the bottom right are classified to represent the unhealthy category. This includes images of burgers, ramen, and spaghetti.

**Figure 3:**
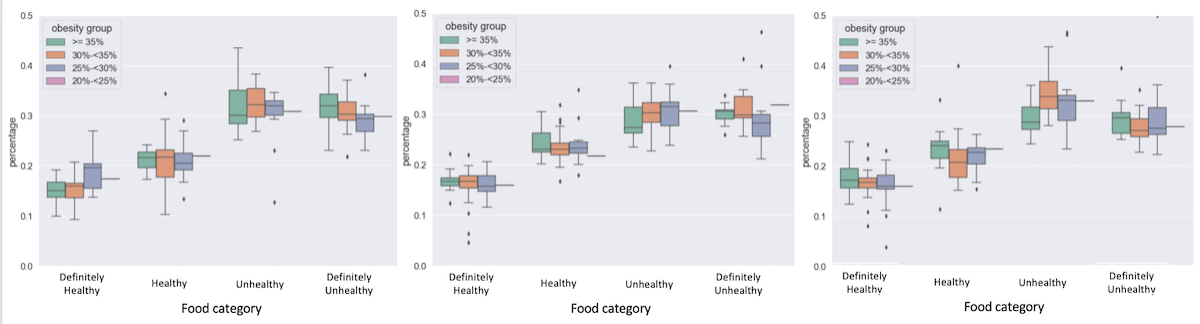
The relationship between the state-level obesity rate and dieting images. The x-axis represents food healthiness categories, and the y-axis shows the distribution of the percentage of food within each category across states in different obesity groups.

We obtained the state obesity rate data from the Behavioral Risk Factor Surveillance System (BRFSS). BRFSS provided the ground truth for the prevalence of obesity via self-reported obesity data among US adults by state and territory in 2019, 2020, and 2021. In Figure 3, we classified 49 states into 4 different obesity levels based on the state obesity rate. New Jersey was excluded from this study because the obesity rate data from BRFSS for New Jersey were missing due to insufficient data. As shown in Figure 3, we used the box plot to visualize the relationship between the state obesity rate and the 4 healthy categories of dieting images.

In 2019, we found a significant positive correlation between the percentage of definitely healthy and definitely unhealthy food in states and the state obesity rate, as measured by Pearson correlation. The percentage of definitely healthy food is negatively correlated with the state obesity rate (*r*=–0.360, *P*=.01), while the percentage of definitely unhealthy food positively correlates with state obesity (*r*=0.306, *P*=.03). In other words, Twitter users in a state with higher obesity rates were more likely to post about definitely unhealthy foods and less likely to post about definitely healthy foods than Twitter users in states with lower obesity rates before the COVID-19 pandemic. We found no significant correlation between the state obesity rate and the percentage of healthy and unhealthy food images from tweets in 2019. In 2020 and 2021, Pearson correlation analysis found no significant association between the healthiness of food images in tweets and state obesity rates. People had a higher obesity rate in 2020 and 2021 compared with 2019. The average obesity rate increased by 0.092 from 2019 to 2020. In contrast, the average obesity rate increased by 1.362 from 2020 to 2021.

### RQ2: How Has the COVID-19 Shutdown Affected People’s Dietary Behavior?

We examined how the COVID-19 shutdown affected people’s dietary behaviors. We found that the percentage of nonfood images decreased in 2020 and 2021 compared with 2019. Table 4 shows that the percentage of nonfood images in 2019 was more than 70%, but it dropped to around 63% in 2020 and 2021. This change is significant based on *Z* test with a *P* value smaller than .01.

**Table 4:** Result of image classification models.

Year	Food, n (%)	Nonfood, n (%)	Definitely healthy, n (%)	Healthy, n (%)	Unhealthy, n (%)	Definitely unhealthy, n (%)
2019	36,168 (29.11)	88,042 (70.88)	5937 (16.42)	7694 (21.27)	11,727 (32.42)	10,810 (29.89)
2020	35,184 (37.24)	59,287 (62.76)	5870 (16.68)	8170 (23.22)	10,928 (31.05)	10,216 (29.03)
2021	31,049 (36.77)	53,401 (63.23)	5360 (17.26)	6850 (22.06)	10,164 (32.74)	8675 (27.94)

We also found that people chose to eat more healthy food during and after the COVID-19 shutdown. The percentage of healthy food (ie, healthy and definitely healthy) increased significantly from 37.69% in 2019 to 39.99% in 2020 (*P<*.001). The percentage of healthy food dropped slightly to 39.32% in 2021, but it was still significantly higher than that of healthy food in 2019 (37.69%).

**Figure 4:**
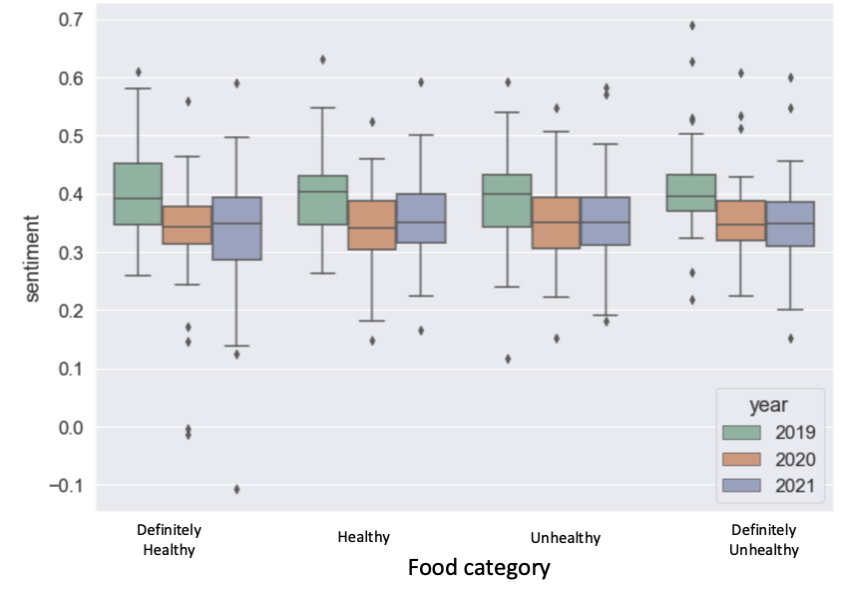
The sentiment to different health categories of food. The x-axis represents food healthiness categories, and the y-axis shows the distribution of the average sentiment within each category across different years.

Another change we saw from Figure 4 is that users showed a significantly more positive sentiment when posting dietary behaviors on Twitter in 2019 than in 2020 and 2021, irrespective of the healthy level of the food. The mean sentiment score for 2019 was 0.395 (SD 0.425), for 2020 was 0.349 (SD 0.432), and for 2021 was 0.348 (SD 0.431). However, the sentiment difference between healthy and unhealthy food was not significantly similar to the RQ1 findings. We conducted *t* tests to evaluate the differences between the 2 groups in each year. The *P* values were .73 for 2019, .24 for 2020, and .68 for 2021.

**Figure 5:**
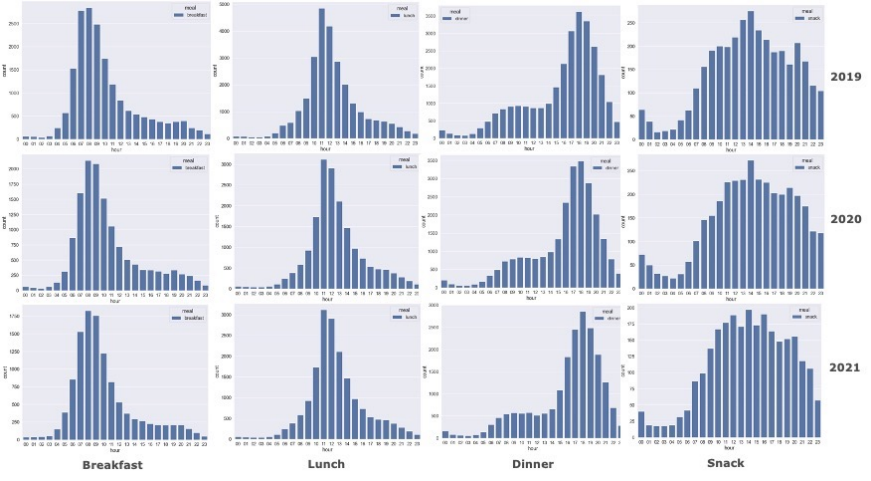
Temporal histograms showing the popularity of breakfast, lunch, dinner, and snack by hour of the day. X-axis of each graph is the hour of the day (0-23). Y-axis is the number of tweets containing that term. In 2020 and 2021, users showed a trend to have breakfast later than and to have dinner earlier than in 2019. People tended to have snacks more often in 2020 and 2021.

We also check the popularity change of different terms: breakfast, lunch, dinner, and snacks. The popularity change of these terms in a day is shown in Figure 5. The COVID-19 shutdown did not change the peak hours of 3 meals: breakfast was mostly mentioned at 8 AM, lunch clustered at 11 AM, and dinner peaked at 6 PM. However, in 2020 and 2021, during and partially after the COVID-19 shutdown, users showed a trend to have breakfast later than usual and to have dinner earlier than usual. In 2019, the most common times for breakfast were 7 AM and 8 AM, and it shifted to 8 AM and 9 AM in 2020 and 2021. The mentioning of dinner was found to be more common at 6 PM and 7 PM in 2019, but it changed to 5 PM and 6 PM in 2020. This changed to encompass the 5-7 PM time window in 2021. We also found that people tended to have snacks more often during and after the shutdown than before. In 2019, the 2 peak hours for snacks were 2 PM and 8 PM. However, in 2020, only one peak hour for snacks was found, which was 2 PM. In 2021, no peak hours for snacks were found.

The approach used to gather the training images resulted in approximately 90% of the images sourced from Google featuring only a single food item. This makes it challenging for the model to do classification when the food in different health categories is mixed up. Future studies should improve this by first identifying the main entree in the image. We can also improve the model’s performance by including more images of more types of food in our training dataset. Our current training schema only contains 40 kinds of food, and some popular food items are not included, such as cheese, noodles, and beans.

We also found that the percentage of definitely healthy food increased for breakfast, lunch, and dinner, while the percentage of definitely unhealthy food decreased for breakfast and dinner. People were also found to have healthier snacks during and after the COVID-19 shutdown. The percentage of definitely healthy snacks increased from 16.6% in 2019 to 19.3% in 2020 and 2021, and the percentage of healthy snacks increased from 21% in 2019 to 24.5% in 2020 and 23.1% in 2021. The percentage of unhealthy snacks decreased after 2019, from 26% in 2019 to around 22% in 2020 and 2021.

To summarize, our study found that individuals’ dietary behavior shifted toward more healthy options during and after the COVID-19 shutdown. This was evident through the selection of more nourishing food options (Table 4) and increased feelings of positivity while making dietary choices (Figure 4).

## Discussion

### Principal Findings

In this paper, we present how the COVID-19 shutdown affected people’s dietary behavior by examining Twitter images and texts collected from 6 different hashtags [27] (#Breakfast, #Lunch, #Dinner, #Brunch, #Snack, #Meal, and #Supper) related to people’s dietary behaviors. Using computational methods, we classified the posted images into different healthiness levels. Our analysis revealed a significant correlation between the percentage of definitely healthy and definitely unhealthy food in a given state and the obesity rates observed in that state in 2019. To gain further insights into how the COVID-19 shutdown affected people’s dietary behaviors, we analyzed collected tweets from associated sentiments with dining and eating patterns. We found that people had a notable positive change in dietary habits by eating more healthy and less unhealthy during and after the shutdown.

The model’s performance on Twitter data did not perform well compared with the training data. This is because the images on Twitter were often more complex than those in the training dataset. For example, Twitter images may have contained multiple food items, or the food item may have not been the focus of the image. For example, in one image, a person was holding a large burger with fries, but the image also showed a small salad on the table in the background. Similarly, another image showed a person working on a laptop with a cup of coffee, and a small cookie was also visible on the desk.

While there are limitations to improving the model’s performance, our analysis highlights the potential of image analysis as a valuable resource for learning about the diet-related daily activities of the general public at the state level [37]. According to our analyses, people’s dietary healthiness is related to the state obesity rate in 2019. Users in states with higher obesity rates post more high-caloric, unhealthy food than healthy food. Dietary healthiness is critical to learn, as it is a leading cause of deaths and increased health care expenditures. It is also related to diet-related chronic diseases [38]. Improving diet quality is becoming more and more vital during COVID-19, because our nutrition intake can affect our body’s ability to fight off infection [39].

The COVID-19 pandemic had a dramatic impact on people’s daily lives, including dietary patterns; we found a decrease in the percentage of nonfood images posted on social media in 2020 and 2021 compared with 2019. This is likely due to a number of factors, including the closure of dine-in restaurants and the social distancing policies that were implemented during the pandemic.

We also observed from Twitter images that the COVID-19 shutdown had some health improvements in dietary healthiness during and partially after the pandemic period. This finding is consistent with previous research [10], a survey study that identified predictors of perceived dietary healthfulness changes over the COVID-19 period in the United States.

To understand the types of healthy and unhealthy foods that are gaining or losing popularity over time, we randomly sampled and manually analyzed 50 images from the unhealthy and healthy categories for each year. The most common subjects for images containing unhealthy food are bakery, dine out, and hot dog in 2019; homemade pizza, ice cream, and cupcakes in 2020; a mix of homemade bakery, stadium (buffet), and catering in 2021. The most common themes for healthy food are fruit, salad (dine-out and homemade), and tacos in 2019; fruit and take-out salad in 2020; and raw/cooked seafood, salad, grilled vegetables, and protein in 2021. Aligned with previous findings [11], our findings reveal that take-out consumption increased, and frozen meals decreased during the pandemic shutdown.

In addition to the positive changes, our study also found some adverse effects of the COVID-19 shutdown on people’s emotions with respect to their dietary behaviors. Consistent with previous studies [30,40], we found the average sentiment to dietary-related tweets is positive. However, the percentage of negative tweets increased by 25% during the COVID-19. This is consistent with studies that have reported that participants experienced moderate to high stress levels during the pandemic period [10,41,42], which can manifest in negative dietary behavior. For example, increased psychological stress is associated with binge eating disorder [43] and reduced diet quality [44], highlighting the need for further explorations.

Contrary to previous studies, users did not express more positive sentiments when eating unhealthy food [45]. To identify factors that affect users’ emotions related to dietary-related behaviors, we used the keyword extraction algorithm term frequency-inverse document frequency to summarize the topics of posts with different sentiments. We analyzed tweets as documents, with each word representing a potential topic. We found that the most common topics of negative tweets during the pandemic were “miss” and “cook”. Here are a few modified examples:

Even as an introvert this quarantine isn’t easy...i miss my friends and hood rat adventures...that said to hold myself accountable I’m committing to 1 bar night and one small gathering a month!

Sadly, I’ve cooked more at home in the last 6 weeks than I probably cooked in the last year. Tonight’s dinner: pork tenderloin, spicy skillet corn, collards and cornbread.

A long shitty day calls for a home-cooked dinner for bae...

Those negative emotions were found to be caused by complaints about current lifestyles, rather than diet-related concerns.

We also found that people’s eating patterns changed during COVID-19, with people eating breakfast and lunch later, dinner earlier, and snacking more often. Changes in eating patterns and dietary healthiness during the COVID-19 pandemic can be explained by lifestyle changes, such as the transition from working in the office to working from home. Additionally, people may be more likely to cook meals at home, which can improve dietary health [4]. Being less exposed to the current obesogenic environment can help people make healthier diet choices [20].

### Implications

Understanding how COVID-19 has influenced dietary behaviors is multi-faceted and significant. By providing a clearer picture of how lifestyle changes affect health, stakeholders can implement more effective interventions at various levels, from individuals to policy makers, thereby ensuring better health outcomes in the face of future public health challenges.

For public health policy makers, insights gained from this study on lifestyle change during the pandemic can inform more nuanced health policies that not only address the immediate concerns about virus transmission, but also long-term health consequences related to diet and obesity. Our findings reveal a correlation between social media food trends and obesity rates. This suggests that public health interventions targeting diet quality could be effectively tailored based on social media trends to address obesity and related health issues in specific regions. For example, based on the current trends, policy makers could consider strategies that encourage restaurants to offer healthier takeout options, promoting users to share their meals on social media, supporting home cooking initiatives to foster a healthier food environment, even under pandemic restrictions.

For individuals, the increase in negative sentiments associated with dietary behaviors during the pandemic highlights the complex relationship between mental health and eating habits. It is crucial to address mental health issues and stress management to promote a healthy eating habit. Another pattern we found for individuals is the shifts in meal timing. The pandemic influenced when people ate, this change could have various health implications. Maintaining a consistent and healthy living routine is essential for sustaining a healthy lifestyle in challenging times.

### Limitations and Future Directions

Our study has some limitations. First, the image classification model could be improved in several ways. We only collected the most popular 10 types of food images for each health category. Our study would benefit from the development and implementation of a more refined food category schema. The updated schema would allow for more precise categorization and analysis of food items, ultimately enhancing the overall quality and impact of our research. Moreover, Figure 2 shows that the health category model’s performance on the healthy and unhealthy categories is not as good as its performance on the definitely healthy and definitely unhealthy categories. In our future studies, we will collect more image data to train the image classification model. For example, we could use ImageNet to train our image classification models. Additionally, our model may not be able to accurately assess the overall healthfulness of a plate of food. In our classification model, we only crop the center of an image to do the classification. However, it is possible for more than one food to be present in the center of an image. Building on our findings, future studies could explore several avenues to further enhance our image classification models. Since our study, new advanced algorithms have been introduced, such as You Only Look Once [46] and Segment Anything Model [47]. Integrating these advanced algorithms could further improve our study’s accuracy and efficiency as they performed well in various image recognition tasks. In addition, a more refined food category schema that contains a wider variety of food would allow for more accurate categorization and analysis of food items, ultimately enhancing the overall quality and impact of our research.

Another limitation of our study is the selection bias of Twitter users and the use of a single social media platform. Only 6.6% of Twitter users were reported to be younger than 18 years [8]. Although our study found that the COVID-19 shutdown had some positive impacts on people’s dietary healthfulness, another study reported its negative effects on children’s dietary healthfulness. A longitudinal study on obese children in Italy found that potato chip, red meat, and sugary drink intakes increased significantly during the lockdown [48]. Future studies should take these limitations into consideration. A more specific user group on social media could be targeted, such as parents of kids, to learn the health-related behaviors of those nonusers.

### Conclusions

In this study, we used social media data to investigate the impact of the COVID-19 shutdown on people’s dietary behaviors. Deep learning image classification procedures and text analysis methods were used to determine that the shutdown had a negative impact on users’ emotions but a positive impact on dietary patterns and sentiments toward them. The findings of this study demonstrate the potential of social media images as a valuable resource for learning about users’ health-related behaviors. The findings could be used as a starting point for further research into the factors that impact users’ dietary behaviors and how COVID-19 affects users’ health outcomes.

## Abbreviations

BRFSS: Behavioral Risk Factor Surveillance System
VADER: Valence Aware Dictionary for Sentiment Reasoning

## Data Availability

The entire datasets generated and analyzed during this study are not publicly available due to the developer policy of X (previous Twitter), but Twitter IDs can be made available from the corresponding author upon reasonable request.
